# The evolution of ageing: classic theories and emerging ideas

**DOI:** 10.1007/s10522-024-10143-5

**Published:** 2024-10-29

**Authors:** Mark T. Mc Auley

**Affiliations:** School of Science, Engineering and Environment, University of Salford Manchester, Salford, M5 4NT UK

**Keywords:** Ageing, Senescence, Longevity, Evolution, Pluralism

## Abstract

Ageing is generally regarded as a non-adaptive by-product of evolution. Based on this premise three classic evolutionary theories of ageing have been proposed. These theories have dominated the literature for several decades. Despite their individual nuances, the common thread which unites them is that they posit that ageing results from a decline in the intensity of natural selection with chronological age. Empirical evidence has been identified which supports each theory. However, a consensus remains to be fully established as to which theory best accounts for the evolution of ageing. A consequence of this uncertainty are counter arguments which advocate for alternative theoretical frameworks, such as those which propose an adaptive origin for ageing, senescence, or death. Given this backdrop, this review has several aims. Firstly, to briefly discuss the classic evolutionary theories. Secondly, to evaluate how evolutionary forces beyond a monotonic decrease in natural selection can affect the evolution of ageing. Thirdly, to examine alternatives to the classic theories. Finally, to introduce a pluralistic interpretation of the evolution of ageing. The basis of this pluralistic theoretical framework is the recognition that certain evolutionary ideas will be more appropriate depending on the organism, its ecological context, and its life history.

## Introduction

Recent years have witnessed significant attention directed towards understanding the mechanisms of ageing and its origins. Indeed, research into the basic processes of ageing is increasingly becoming an accepted pillar within mainstream biomedical research (Mc Auley et al. 2017; Morgan and Mc Auley 2020; Keshavarz et al. 2023; Marx 2024). This contrasts sharply with previous circumstances, where biogerontologists operated on the margins of biomedical science. Individuals who now opt for a career in ageing research follow pioneering work. Nowhere better exemplifies this than the evolutionary biology of ageing, a subject which some of biogerontology’s most significant trailblazers have devoted considerable time to (Comfort 1964; Partridge and Barton 1993; Rose 1994; Hayflick 1998; Kirkwood 2005; Finch 2010; Kenyon 2010). Understanding the evolution of ageing requires a knowledge of Darwinian biology. It also necessitates conceptual clarity around two key biogerontological terms, which are relevant to this critical review. The first is senescence. Many definitions of senescence exist. However, a broadly accepted view from a life-history perspective is that senescence is the physiological deterioration of an organism that results in declining survival and/or reproduction with age (Stearns 1998). The second term is ageing, which is also challenging to define. Despite this, most biogerontologists would agree that ageing is the cumulative impact of a series of deleterious biological processes which gradually renders an organism increasingly vulnerable to mortality with age (Mooney et al. 2016; López-Otín et al. 2023). It is apparent ageing and senescence are different. The reason for presenting this distinction is that in the biogerontology literature they are often conflated. In this review each will be explicitly referred to and the terms will not be used interchangeably to mean the same thing. This is particularly important when discussing evolutionary processes where changes in Darwinian fitness carry significant conceptual meaning.

Weismann first adopted Darwinian logic to understand ageing. He reasoned it evolved to remove older individuals from a population, thus freeing resources for younger group members (Weismann 1891; Kirkwood and Cremer 1982). Weismann viewed ageing as an adaptive trait beneficial to the species rather than individuals. However, evolution is now understood to act at the level of genes, not populations or individuals (Dawkins 1976; Williams 2018; Ågren 2021). The notion of ageing as an adaptive process did not die with Weisman; ideas of this nature persist (Pamplona et al. 2023). However, most theories regard ageing as a non-adaptive by-product of evolution which is due to a decline in the force of natural selection. This premise underpins the mutation accumulation (MA) (Medawar 1952), antagonistic pleiotropy (AP) (Williams 1957), and disposable soma (DS) (Kirkwood 1977) theories. These three ideas constitute the classic evolutionary models of ageing. Empirical evidence exists which supports each classic theory (Mc Auley 2021). However, it is important to note that this support is based largely on qualitative observations regarding processes such as the intensity of selection. Thus, a consensus remains to be fully established as to which theory best accounts for the evolution of ageing. This uncertainty has led to the development of arguments which advocate for alternative theoretical frameworks, some of which propose an adaptive origin for ageing, senescence, or death (Galimov and Gems 2021; Pamplona et al. 2023). Other work has critically dissected the classic theories, or attempted to reinvent/unify them (Johnson et al. 2019; Lemaître et al. 2024). In contrast this review will evaluate how mechanisms beyond a unidirectional decline in natural selection affect the evolution of ageing. Moreover, I critically examine alternatives to the classic evolutionary theories, and pay particular attention to how the concept of evolutionary entropy (EE) relates to MA, AP and DS. The review concludes by introducing a pluralistic interpretation of the evolution of ageing.

## The classic evolutionary theories of ageing

The foundations for the classic theories were laid by Fisher and Haldane, who understood that natural selection has a stronger impact on reproduction and survival in early years than it does later in life (Fisher 1930; Haldane 1942). This concept underpins the MA theory, which suggests ageing is due to the accumulation of harmful alleles, which escape natural selection, due to only being functionally expressed post reproductively (Medawar 1952). AP is an extension of MA. It proposes that alleles have antagonistic effects, which are beneficial during early life but are detrimental in later life (Williams 1957). According to AP the positive effect on fitness of an allele outweighs its negative effect, due to the greater intensity of natural selection early in life. Formal mathematical credence was given to the idea that natural selection declines with age by Hamilton and others (Hamilton 1966; Rose 1994; Charlesworth 2000). And this principle was further used as the basis of the DS, underscoring that MA, AP and DS are not mutually exclusive concepts. However, subtle differences do distinguish DS from the other two theories. DS is a physiological idea suggesting ageing evolved as a result of the strategic partitioning of energy between somatic maintenance and reproduction (Kirkwood 1977; Kirkwood and Holliday 1979). Energy is allocated based on the life expectancy of the organism. Life expectancy is in turn moulded by parameters that influence extrinsic mortality, such as predation rate. The DS theory implicitly assumes that sufficient energy will be invested in somatic maintenance so that an organism reaches reproductive years, but it is not an optimal strategy to invest in somatic maintenance indefinitely if the rate of extrinsic mortality dictates that an organism will not live much beyond reproductive age. Conversely, intrinsic mortality is driven by life history decisions such as how much resources is allocated to reproduction.

## Empirical support for the classic ageing models

An evolutionary theory must be evaluated based on its success in explaining observed biological phenomena. This test has been largely passed by the classic theories as they are empirically supported. It is beyond the scope of this review to discuss the evidence in depth. This has been dealt with previously (Johnson et al. 2019; Mc Auley 2021). However, it is worth mentioning some recent studies which support the classic theories. A recent investigation provided some compelling evidence for MA by demonstrating how accurate mutation accumulation rate is between diverse species (Cagan et al. 2022). Recent support for AP derives from an *Apolipoprotein-ε4 (APOE-ε4)* allele study (Trumble et al. 2023). *APOE* is crucial to lipid metabolism (Yang et al. 2023). However, the *APOE-ε4* allele is associated with increased risk of Alzheimer’s disease, increased risk of cardiovascular disease and decreased lifespan (Belloy et al. 2019). In the study by Trumble and colleagues it was observed that within a population of 795 women forager-horticulturalists (the Tsimane) those who possessed two copies of the *APOE-ε4* allele gave birth to more children than women with other allele combinations. This is consistent with the idea that *APOE-ε4* is an AP gene*.* In the case of the Tsimane an increased risk of age-related morbidity and mortality is the evolutionary cost for increased fecundity. Some recent findings consistent with DS logic derive from canine investigations. In one study a reciprocal relationship was observed between reproductive investment in dogs and their lifespan (Bargas-Galarraga et al. 2023). Further work observed an inverse relationship between life span and body size, with the authors arguing that the findings are conceptually close to DS (da Silva and Cross 2023).

## Conceptual and empirical challenges to the classic models

As alluded to ample empirical support exists for the core elements of the classic theories. However, several conceptual and empirical challenges have emerged that question aspects of these theories. Firstly, both MA and AP treat ageing as the cumulative manifestation of late acting alleles. This is a satisfactory explanation for explaining why certain alleles are associated with age-related diseases (Byars and Voskarides 2020; Lockwood et al. 2024). However, AP and MA distil ageing to essentially a genetic problem. Unfortunately this ignores the malleability of ageing, which has been experimentally demonstrated in a wide variety of organisms (Hwangbo et al. 2020; Keshavarz et al. 2023). Some empirical evidence is also difficult to reconcile with the classic theories. For instance, MA may not be a significant cause of ageing. This is evidenced by work in *saccharomyces cerevisiae* which has revealed that the number of mutations which accumulate with age are of an insignificant level (Kaya et al. 2015). Moreover, in some studies when mice were genetically manipulated to have high mutation rates this did not unduly impact their lifespan (Narayanan et al. 1997). On a similar note, in some studies mice subjected to a high level of free radical induced somatic damage do not suffer from a significant reduction in lifespan (Van Remmen et al. 2003; Lapointe and Hekimi 2008). Findings which are at odds with the notion that somatic impairment drives ageing, something which is a core feature of DS. It is worth exploring in depth how some other factors present a challenge to the classic theories.

### Darwinian fitness

The classic models are built on the premise that population growth rate is the measure of Darwinian fitness (Charlesworth 2000). This parameter does not take into consideration factors such as population size and ecological constraints (Dietz 2005). This limitation has its origins in the quantitative work of Fisher and his treatment of the Euler-Lotka equation (Fisher 1930). Fisher used this equation to represent an age-structured population reproducing in continuous time. He suggested that such a group of individuals will reach an asymptotic exponential rate of population increase, which is denoted as, *r*. Fisher referred to *r* as the Malthusian parameter of the population, and he proposed it to be the key measure of Darwinian fitness (Fisher 1930; Rose 1994; Charlesworth 2000). This way of defining Darwinian fitness is the foundation of classic evolutionary thinking (Charlesworth 2000). However, the notion of *r* has been challenged analytically (Demetrius 2001). Based on this analytical work it is suggested that the rate at which a population returns to its original size after a random perturbation in the age-specific birth and death rates is the key determinant of Darwinian fitness (Demetrius 2001; Olshansky and Rattan 2005; Demetrius et al. 2009). It has also been suggested that the rate at which a population returns to a steady state can be quantified by a demographic parameter, denoted as EE. In other words EE quantifies variability in the age individuals reproduce (iteroparity) and die (Demetrius et al. 2009). EE was recently used to account for the diversity of ageing patterns across the tree of life (Buescu et al. 2023). Using EE as a measure of Darwinian fitness it was predicted that natural selection can be strong in both early and late life (a convex function). This contrasts sharply with the monotonic decline in natural selection which is the cornerstone of the classic theories (Flatt and Partridge 2018). This prediction is feasible as stochasticity is crucial to demographic processes, and EE is capable of representing it as a key determinant of Darwinian fitness.

### Extrinsic mortality an ongoing debate

A central tenet of the classic theories is that an increase in extrinsic mortality results in the evolution of a faster ageing rate (Caswell 2007; Dańko et al. 2018). In fact, the close coupling of extrinsic mortality with life-span variation has been consistently observed throughout nature (Reznick et al. 2002, 2004). Moreover, differences in extrinsic mortality are suggested to account for lifespan plasticity among diverse species (Wilkinson and South 2002). A common example is birds where key features of their behaviour, and life history are associated with reduced extrinsic mortality rates, when compared to mammals of a similar size (Holmes and Austad 1994, 1995; Wasser and Sherman 2010). An explanation for this phenomenon is that birds have a decreased rate of ageing compared to similar sized mammals, as flight lowers their predation rate (Pomeroy 1990). Similarly, it has been suggested that arboreality offers a protected environment which abrogates predation exposure, reducing extrinsic mortality and favouring the evolution of increased lifespan (Shattuck and Williams 2010). Indeed, protective phenotypes (e.g. tortoise shells, arboreality, fossoriality etc.) were recently identified as key determinants of lifespan in tetrapods, in particular they were highlighted as particularly important to the evolution of longevity in bats, birds and naked mole rats. (Shilovsky et al. 2024).

It is crucial to note that a reduction in predation has not been universally observed to result in decreased ageing in all organisms (Pietrzak et al. 2020). Moreover, despite the empirical observations theoretical work has suggested that under certain conditions a higher rate of extrinsic mortality results in the evolution of slower senescence (Abrams 1993; Williams and Day 2003; André and Rousset 2020). Moreover, it has been argued that for extrinsic mortality to impact senescence it needs to do it in an age dependent way (Moorad et al. 2019). Although this notion has been strongly contested (Day and Abrams 2020). Despite this it is acknowledged that in certain species population density dependence introduces an additional layer of complexity which makes determining how extrinsic mortality influences senescence particularly challenging (Day and Abrams 2020). This line of thinking is supported by recent thought-provoking work which convincingly argues that the effects of extrinsic mortality are closely tied to density dependence (de Vries et al. 2023). Intriguingly, this work suggested that depending on the specific circumstance a change to extrinsic mortality may not result in faster/slower lifespan. The impact of considering density dependence was further emphasised recently when mathematical modelling was used to explore MA theory using a hypothetical population. The model predicted that density-regulated populations will end up semelparous or collapse due to the accumulation of deleterious mutations (Aubier and Galipaud 2024). This finding appears to reveal an inherent weakness of classic evolutionary thinking and in particular MA.

### Negligible or negative senescence

The concept of negligible or negative senescence is based on observations in certain species which have revealed that risk of mortality may not change, or even decrease with chronological age (Finch 1994; Vaupel et al. 2004). For instance, naked mole rats display virtually no increase in mortality risk with age, possess a delayed ageing phenotype throughout their life-span, and show resistance to age associated pathologies, such as cancer (Oka et al. 2023). Moreover, in a recent investigation involving turtles and tortoises who were kept in zoos it was observed that ~ 75% of 52 species exhibit slow or negligible senescence (da Silva et al. 2022). Interestingly, however, when negative senescence was quantitatively interrogated recently using mathematical modelling, it was found that the magnitude of mortality increase has been significantly underestimated based on reported lifespans (Xia and Møller 2022). However, if the assumption is made that most observations of negligible/negative senescence are reliable, this generates a theoretical conundrum for the classic theories. Negligible/negative senescence is inconsistent with the notion that the diminishing capacity of natural selection with advancing age favours trading-off late-life fitness for early reproductive success. Although a solution may exist as to why selection does not decline with age in organisms that manifest negligible/negative senescence. Recent comparative work emphasized that population structure and reproductive value are crucial to interpreting selection gradients (Roper et al. 2021). This study revealed how growth form, individual trade-offs, stage structure and social interactions influence various distributions of population structure and reproductive value. It was contested such factors have the potential to help understand why patterns of negligible/negative senescence could be accounted for under the same evolutionary models as ‘normal’ senescence.

### Sexual selection and ageing patterns

Sexual selection influences natural selection. This impacts the classic theories because they are built on the assumption that ageing evolved under natural selection. However, unlike natural selection which results from differential fecundity and survival, sexual selection is the outcome of variance in mating/fertilization success (Trivers 1985; Rowe and Rundle 2021). Typically the strength of sexual selection is stronger in males than females (Borgia 1985; Emlen et al. 2005; Clutton-Brock 2007; Dakin and Montgomerie 2013). Moreover, in certain organism’s males invest less in generating offspring than females (Trivers 2017). In theory this means males can direct additional resources to attracting females. Moreover, females can then choose a mate based on the effort’s males direct towards their sexual fitness (Shuker and Kvarnemo 2021). This may necessitate a male reproductive strategy which deals with inter-male rivalry/conflict (Le Boeuf 1974; Simmons and Emlen 2006).

In many species sexual selection mediates how males and females senesce (Carranza et al. 2004; Gasparini et al. 2019). Indeed in several species males that invest more strongly in traits which help them win conflicts, or which make them more attractive to females benefit from increased reproductive success (Bagchi et al. 2021). The drawback is that male reproductive strategies can be associated with an increased risk of extrinsic mortality, and thus a shorter lifespan (Bonduriansky et al. 2008). Indeed, in certain species male-male competition for early reproductive success can result in differential rates of senescence (Preston et al. 2011). Males may also possess traits that make them more attractive to females as their age increases (Lifjeld et al. 2022). Such instances potentially lead to the selection of alleles which favour male fitness and longevity (Brooks and Kemp 2001).

Female lifespan is also influenced by sexual selection. For instance, females may evolve ornamental features to attract a suitable mate. Such ornamentation requires resource investment which may influence female life-history schedules (Potti et al. 2013). Moreover, females can evolve sexual features which directly influence their longevity (Møller 1993). Of course, sexual conflict may also impact the evolutionary relationship between males and females. Sexual selection can ‘drive’ a male trait beyond its naturally selected optima (Hosken and House 2011). This demonstrates that sexual selection can antagonistically oppose natural selection (Kirkpatrick 1982). Female fitness can also be impacted by male sexual selection which in turn can influence their lifespan. For instance, female *Drosophila melanogaster* which possess longer sperm receptacles (SR) have shorter lifespans (Miller and Pitnick 2003). This is thought to be a result of sexual conflict mediated by ejaculate toxicity. More recent work has found that male and female flies which possess giant sperm and long SR genotypes have increased longevity (Zajitschek et al. 2019). Examples also exist in many species, including humans, where certain females prefer to mate with older males (Côté and Hunte 1993; Kokko and Lindström 1996; Brooks and Kemp 2001; Conroy-Beam and Buss 2019). This phenomenon could exist because older males have proven their capacity to survive, and provide females with access to resources (Kokko and Lindström 1996). Thus, sexual selection can drive a pro-longevity phenotype. However, an evolutionary cost can be associated with mating with older males. Recent epigenetic inheritance studies have observed that mating with an older male can lower female fecundity and reduce offspring fitness (Mc Auley 2023). Such observations add further intrigue to interpreting the eco-evolutionary dynamics of sexual selection and its relationship with the classic ageing theories.

### Genetic drift and gene flow

Genetic drift, founder effects, and gene flow are important evolutionary forces that have contributed significantly to evolution (Simon and Coop 2024). Genetic drift is recognised as one way in which isolated human populations such as those in Okinawa and elsewhere may have developed extreme longevity phenotypes (Bendjilali et al. 2014). However, genetic drift is not universally associated with the evolution of longevity phenotypes. In *Daphnia magna*, it has been observed that genetic drift is synonymous with an increased ageing rate and a reduction in lifespan (Lohr et al. 2014)**.** Of course, genetic drift intersects with other evolutionary forces such as sexual and natural selection. Species who practice sexual polygamy are suggested to be more impacted by genetic drift (Charlesworth 2009). Moreover, longevity in polygamous species could be counteracted by the persistence of deleterious alleles which are maintained by sexual selection because of their benefit to the other sex (Arnqvist and Rowe 2005). Gene flow into a population can oppose gene frequency changes; conversely it introduces genetic variation, which is the substrate for evolution by natural selection (Lenormand 2002). However, restricting gene flow out of a population has evolutionary benefits. A narrow niche breadth (NB) increases the likelihood of fixing beneficial alleles, reduces the frequency of deleterious alleles drifting to fixation, and diminishes mutation load (Whitlock 1996). However, interestingly from the perspective of the classic theories an increase in population density can result in a decrease in NB (Nicholson et al. 2006), while changes to NB can impact lifespan (Roff 2002; Jervis et al. 2007).

### The effects of dietary restriction

Empirical evidence in a range of organisms over the last number of decades has identified dietary restriction (DR) as a robust intervention for extending both health-span and lifespan in many model organisms (Duan et al. 2024). It remains unknown how the effects of DR reconcile with the classic theories (Moatt et al. 2020). What is known however is that a key feature of the classic theories is that they are underpinned by a trade-off between survival and reproduction. This trade-off has been used as the basis of an evolutionary explanation for the pro-longevity effects of DR. It is parsimoniously suggested that there is a metabolic shift towards somatic maintenance at the expense of reproduction (Kirkwood and Shanley 2005). This idea is known as the resource reallocation hypothesis (RRH), and it is theoretically underpinned by DS (Shanley and Kirkwood 2000). Evidence exists which is consistent with the RHH (Mc Auley 2022). However, the intricacies of this relationship are not completely clear. This is evidenced by a recent study which found that fasting increased investment in the soma of zebrafish upon refeeding at the expense of gamete quality (Ivimey-Cook et al. 2023). Other, studies have suggested that during DR in some organisms the trade-off between survival and reproduction can be uncoupled by altering the intake of different dietary nutrients such as cholesterol, amino acids, and carbohydrates (Grandison et al. 2009; Zanco et al. 2021). This has led to alternative evolutionary ideas to RHH (Adler and Bonduriansky 2014; Speakman 2020). A further conceptual puzzle for RHH centres on studies which have shown that DR can have inter- (one generation) or trans-generational (multi-generational) effects on survival and fitness (Ivimey-Cook et al. 2021; Camilleri et al. 2024; Jimenez-Gonzalez et al. 2024). Thus, DR and its intersection with epigenetic inheritance have yet to be satisfactorily reconciled with the classic theories.

### Can non-genetic inheritance be reconciled with ageing evolutionary theory?

Biogerontology is being significantly influenced by progress in epigenetics (Mc Auley et al. 2018; Morgan et al. 2018, 2020; Larson et al. 2019; Zagkos et al. 2021; Seale et al. 2022; Phyo et al. 2024). DNA methylation (DNAm) clocks have necessitated a revaluation of the proximate causes of ageing (Horvath 2013; Zheng et al. 2024). Moreover, emerging findings from epigenetic inheritance (EI) studies have stimulated debate about the ultimate cause of ageing (Woodhouse and Ashe 2020; Ghai and Kader 2022). EI implies that non-genetic mechanism(s) could influence evolution. It must be emphasized however, that EI is controversial; and it remains to be fully established as to how it reconciles with Darwinian theory let alone the classic evolutionary models of ageing. Some recent work did however, address this burgeoning theoretical conundrum (Mc Auley 2023). It was broadly concluded that the examples of EI and its impact on offspring longevity mainly derive from canonical organisms such as worms and flies. Furthermore, some evidence was highlighted that implies epigenetic patterns can be transmitted to gametes from the soma. Although, it is important to reiterate that the evidence for EI and its potential role in the evolution of ageing is very much an open question for biogerontology despite the emergence of some intriguing recent findings.

### Social modulation of ageing evolution

The concept of inclusive fitness (IF) helps explain social behaviour from an evolutionary perspective (Hamilton 1964). Arguably, however, IF has yet to be fully reconciled with the classic evolutionary theories. Indeed, when Hamilton predicted that the force of natural selection decreases with age, this work did not include IF (Hamilton 1966). Rather the model included general fitness as a parameter and did not consider IF as a contributor to the moulding of senescence. This omission is significant because arguably the 1964 work of Hamilton is the foundation which is used to support the main premise of the classic theories. Consequently the classic theories are deficient at describing how species-specific ageing patterns evolve in social organisms (Korb and Heinze 2021). A central issue is that in certain species sociality is associated with a reversal of the suggested trade-off between longevity and fecundity which features prominently in classic thinking about how ageing rates evolve (Page and Peng 2001; Blacher et al. 2017; Cohen et al. 2020).

Social populations experience age-associated selective pressures which act on social phenotypes. For instance, in certain species later-life reproductive senescence is suggested to be due solely to the effect of population composition (Rodrigues 2018). Such findings make reconciling sociality with the classic theories problematic. However, some worthwhile attempts have been made to integrate sociality with the classic theories. Ideas have primarily focused on eusocial organisms such as termites, wasps, ants, bees, and naked mole rats (Bourke 2007; Lucas and Keller 2020; Korb and Heinze 2021). Using the specialized morphological castes which is synonymous with these animals it has been argued that unlike workers, queens are shielded from external dangers, consequently natural selection optimizes their fitness into advanced age (Keller and Genoud 1997; Rueppell et al. 2015). Some evidence for this concept derives from the termite *Reticulitermes speratus,* where it has been observed that workers suffer from greater oxidative damage than queens (Tasaki et al. 2018). This phenomenon may not hold for all eusocial species, as queens and workers in naked mole rats do not display significant differences in lifespan, even if queens have been determined to have a slower epigenetic age (Horvath et al. 2022). However, another way to reconcile eusocial insects with the classic evolutionary theories centres on viewing them as a superorganism. According to this idea infertile workers are deemed ‘disposable’ (akin to a soma), because of their decreased lifespan, while the queen is viewed as the germline because she is protected (Rascón et al. 2012). However, insect species such as *Temnothorax rugatulus* exhibit caste plasticity, whereby workers who become fertile express genes which increase their lifespan (Negroni et al. 2021). Thus, the superorganism analogy does not align with the behaviour of all eusocial species.

Mathematical modelling has been used to examine sociality. One study predicted that restricted dispersal and social interactions indirectly produces AP patterns which affects survival and fecundity at different ages (Ronce and Promislow 2010). Another model was based on the assumption that AP mutations operate within or between castes (Kreider et al. 2021). According to this framework antagonistic effects originated due to caste antagonism, or indirectly due to genetic influences between castes. The model predicted that mutations with antagonistic fitness effects within castes reduced the lifespan of both castes. In between caste mutations decreased worker lifespan to a greater extent than queens. It was concluded this undermines the role extrinsic mortality has been afforded in the caste‐specific ageing of eusocial organisms. It was also posited that AP effects castes differentially due to reproductive monopolization by queens. Other work investigated cooperative breeding and ageing (Kreider et al. 2022). Cooperative breeders do not have castes. They temporarily forgo reproduction to become helpers. Simulations predicted natural selection favours delaying senescence in cooperative breeders. It was reasoned they delay age of first reproduction because helpers wait to become a breeder. It was also predicted that reduced genetic relatedness causes the evolution of longer lifespans. This was explained as selection being weaker against higher mortality when death decreases competition for breeding between relatives.

### Intergenerational resource transfer

Intergenerational resource transfer could influence the evolution of ageing. This phenomenon is not part of the classic theories. However, a solid rationale exists for its inclusion, as intergenerational transfers of adult surplus, information, and pedagogy can indirectly contribute to offspring fitness (Gurven et al. 2012; Davison and Gurven 2022). Modelling has also proved useful at exploring this phenomenon (Lee 2003). In a recent study it was used to represent transfers between age classes as the per capita net contribution of all helpful or harmful social behaviours (Roper et al. 2023). By embedding this theoretical adjunct to a model of IF it was revealed that the force of selection on age-specific reproduction does not always decline monotonically from birth, but instead depends on the balance of costs and benefits of increasing reproduction to both direct and indirect fitness.

### Stochasticity and the evolution of ageing

It is possible that stochasticity has influenced the evolution of ageing (Mc Auley and Mooney 2015). This is underscored by recent work which concluded that individual variability was responsible for different ageing patterns in the long-tailed tit *Aegithalos caudatus* (Roper et al. 2022). Further work emphasises how stochasticity can influence the evolution of differences in lifespan. It was observed that genetically identical female *Drosophila melanogaster* that live long, where found to produce long lived daughters, but generated less offspring (Drake and Simons 2023). It was suggested this was due to an apparent trade-off which was solely the result of stochastic effects. It was also suggested that trade-offs such as the one between reproduction, and lifespan may be less widespread than predicted by evolutionary biology of ageing and may instead stem from stochasticity rather than the partitioning of investment between reproduction and somatic maintenance. Fluctuations in the environment also effect evolution. Phenotypic plasticity is the evolutionary adaptation which allows an organism to respond to environmental heterogeneity (Kawecki and Stearns 1993; McHugh and Burke 2022). Phenotypic plasticity is also known to effect longevity in animals (Borges 2009). This makes sense because an inflexibility genome is not very useful, particularly if an animal lives in a fine-grained environment.

### Do the classic theories account for senescence in *bacteria* and plants?

The classic theories provide satisfactory arguments for the evolution of ageing and age-related disease in eukaryotic, iteroparous species. Arguably however, they do not account for senescence in plants and bacteria. This could be significant because some evidence suggests plant senescence has its origins within programmed cell death which is a feature of primaeval unicellular photoautotrophs (Bhattacharjee et al. 2024). Plant senescence is generally regarded to be programmed and the mechanistic underpinning of this phenomenon requires elucidation (Thomas et al. 2009; Thomas 2013). Likewise, the evolution of ageing in bacteria needs further exploration. This assertion is consolidated by experimental work which suggests bacteria are a worthwhile comparative model for studying ageing (Florea 2017; Łapińska et al. 2019; Steiner 2021). For instance, some bacteria exhibit a trade-off between survival and proliferation in a subtle balancing act between reproduction and longevity (Abram et al. 2021). This is similar to the trade-off for resources some higher organisms devote to reproduction/growth versus that allocated for cellular maintenance, which is central to the DS theory (Hammers et al. 2013). Bacteria can also undergo programmed cell death, during nutritional stress (Nyström 2003). Thus, it is possible ageing may have evolved differently in bacteria from other species, nonetheless it is worth examining it, if only for comparative reasons.

## Alternative evolutionary ideas

### Gerontogenes, vitagenes and homeodynamics

Many alternatives to the classic paradigms exist. Some ideas have persisted while others have not. Recently it was argued Weismann’s idea was “mostly correct”, and it is only necessary to reconsider how adaptive ageing originated (Melkikh 2023). Such arguments are perhaps unsurprising given empirical data continues to emerge from canonical organisms, which make this line of thinking conceptually attractive (Kern et al. 2023). For instance, it has been argued that adapted death has evolved in certain organisms which display a high degree of relatedness (Galimov and Gems 2021). Similarly it has been suggested that ageing can be positively selected when kin selection and directional selection are closely coupled (Szilágyi et al. 2023). Another recent proposal theorized ageing evolved due to selection reducing the environmental risk of death (Omholt and Kirkwood 2021). It was posited natural selection could create this adaptation, by using a genetic programme that ensures somatic maintenance remains low.

Theories of programmed ageing depend on the selection of genes for senescence. Clearly such ideas run counter current to the general consensus that ageing is a non-adaptive by product of evolution. However, this does not mean genes aren’t involved in the regulation of senescence, despite not being directly selected for the purpose of ageing. Rattan refers to these genes as virtual gerontogenes (Rattan 1985). To understand how gerontogenes fit with evolutionary theory it is necessary to introduce the essential lifespan (ELS) of a species. ELS is the time needed by a species to grow, develop, mature and reproduce (Rattan 2018). Accordingy to this framework evolution has selected for longevity assurance genes (LAGs) that determine the ELS of a species (Rattan 2018). It is suggested gerontogenes are vital for maintaining germ line continuity as hypothesised by both AP and the DS theories (Rattan 1995).Crucially, Rattan eloquently and convincingly emphasises that it is important not to conflate gerontogenes with genes associated with the heritability of lifespan, such as those described in twin studies, those attributed to premature ageing syndromes, or those genes which have been identified in canonical organisms that result in extended life-span (Rattan 2024). The key point being is that it cannot be convincingly argued that these are gerontogenes which were selected by evolution to cause ageing or death (Rattan 2024).

It is vital to acknowledge that ageing is likely to be an emergent property of a species. Within this line of thinking, ageing can be viewed as a complex network of LAGs. Rattan refers to these as vitagenes (Rattan 1998). The concept of vitagenes also relies on homeodynamics. Homeodynamics attempts to bridge the gap between physics and biology (Yates 1994). It stipulates that biological processes are in a state of dynamic equilibrium, constantly undergoing flux. Adaptation and flexibility are key to homeodynamics, ensuring effective responses to perturbations. Homeodynamics involves non-linear mechanisms where small perturbations can lead to significant changes involving non intuitive, multilevel, feedback processes which give rise to emergent behaviour. Pathways considered part of homeodynamics, centre on maintenance processes, including those involved in DNA repair, heat shock, antioxidant defence, apoptosis, and removal of protein damage. It is theorized optimal homeodynamics is essential for an organism's growth, development, and maturation (Rattan 2018). According to Rattan the homeodynamic property of a biological system is a function of the vitagene network (Rattan 1998). It acts as a longevity assurance process which is moulded by the evolutionary history of the species(Rattan 2007). Homeodynamic space is an extension of this idea, it includes both adaptive homeostasis, and allostasis (Rattan 2007). Homeodynamic space emphasizes the importance to survival and longevity of stress response induced maintenance and repair, damage regulation rate, and immune remodelling, which are defined by the evolutionary history of the organism.

### Quasi programmed theories which centre on growth

It has been proposed that ageing evolved due to a quasi-programme which is underpinned by growth which extends into adulthood. This has been referred to as the hyperfunction theory (HFT) (Blagosklonny 2006; Gems 2022). According to HFT growth continues due to the hyperfunctioning of signalling pathways. This makes some sense because many species that display deterministic growth (growth to a specific adult size) have been observed to senesce, while species displaying indeterminate growth have been associated with negative senescence (Vaupel et al. 2004). The converse of hyperfunction is hypofunction. Hypofunction is speculated to occur if a developmental pathway is shut off /under expressed in later life (de Magalhães and Church 2005). Collectively hyperfunction and hypofunction have been referred to as the developmental theory of ageing (DTA) (Lemaître et al. 2024). Recently an attempt was made to unite the classic theories with DTA (Lemaître et al. 2024). It was posited that both hyperfunction and hypofunction are equally possible avenues for the evolution of ageing. Development is key some other emerging ideas. A recent hypothesis suggests evolution has a bias which is directed towards introducing innovations at the end of development (Fontana and Kyriazis 2024). Known as the evolvable soma theory it postulates that ageing is beneficial as it provides a period whereby innovations can be tested during a time where their impact on the fitness landscape is less significant. Another hypothesis which focuses on growth suggests negative senescence is a fitness neutral trait which evolved as a byproduct of a more important adaptation (Gems and Kern 2024). This is based on the notion of spandrels (Gould and Lewontin 1979; Gould 1997). It suggests AP, biological constraint and associated “quasi-programming” cause age related disease (spandrels). Indeed, other work has suggested ageing is a spandrel. A comprehensive literature survey concluded that in certain species ageing may have evolved due to a trade-off between successful land colonisation and longevity (Bilinski et al. 2021).

### Extended phenotypes and the evolution of ageing

The concept of an extended phenotype hypothesis as introduced by Dawkin’s, suggests an organisms’ genes exert their influence beyond their immediate biological boundaries (Dawkins 1982). An example is the gut microbiome, where it has become increasingly recognized that the microbiome is a pivotal determinant of host phenotype (Dapa et al. 2023). This reveals a key limitation of the classic theories as they do not consider how animal–microbiome interactions have influenced the evolution of ageing. This requires addressing in the future because considerable interest has been generated recently as to the role the microbiome has played in the evolution of humans and other species (Fellows Yates et al. 2021; Dapa et al. 2023). Interestingly, however there has been some speculation as to how microorganisms have influenced the evolution of ageing. An idea known as the age-distorter hypothesis (ADH), suggests that ‘age distorters, including viruses, microorganisms, or multicellular organisms affect host ageing for their own evolutionary benefit. This is an intriguing perspective given that changes to the gut microbiome/virome have been revealed to have a significant impact on the trajectory of human health (Cao et al. 2022; Salvadori and Rosso 2024).

## Towards a pluralistic view of the evolution of ageing

It is undeniable certain challenges remain difficult to reconcile with the classic theories. One solution to this problem is to view the evolution of ageing pluralistically (Mc Auley 2022, 2023). To do this, it is necessary to understand what pluralism is. Pluralism can mean many things, however, in this context pluralism is best thought of as the acknowledgement that many different systems or approaches are appropriate for understanding complex problems (Ruthenberg and Mets 2020). Pluralism has been successfully used to reappraise the tree of life hypothesis (Doolittle and Bapteste 2007). In this context Doolittle and Bapteste define pluralism as: “the recognition that different evolutionary models and representations of relationships will be appropriate, and true, for different taxa or at different scales or for different purposes”. This definition could equally apply to the evolution of ageing. Crucially it underscores that an evolutionary model is context dependent. This perspective is inconsistent with the conventional notion that a single theory exists which can explain different patterns of ageing across all taxa. This is particularly relevant given that the intensity of ageing, and its trajectory appear to be context and taxa specific (Lemaître et al. 2020). This also applies to emerging theories. Nascent ideas contribute to the discourse on the origins of ageing, however arguing that a new theory completely supersedes the classic theories is inadvisable. Thus, this pluralistic approach does not represent a testable theory of ageing in itself but it alters the way we perceive the existing evolutionary theories of ageing, and it may provoke us to conceive different ways of exploring ageing that are not possible to do by restricting ourselves to one particular evolutionary theory. This is akin to Dawkins idea of the Necker cube (Dawkins 1982).

It is necessary to reiterate again that the pluralistic framework espoused here is not an effort to amalgamate the classic theories. It is the opposite, and contrasts with recent work which attempted to integrate the classic theories by examining resource allocation strategies across a range of taxa (Suvorov 2022). It also differs from recent work which suggests the evolutionary theories need to be expanded to better account for symbioses and interactions throughout the web of life (Bapteste et al. 2023). Indeed, there are instances where the classic theories need to be adjusted to account for scenarios, however in most instances, it should be possible to explain an observation using one of, or a combination of the classic theories. Moreover, according to this interpretation of pluralism it is unlikely that a general framework, which describes the evolution of ageing can be identified for all organisms based on the integration of several theories. This does not mean however that some generalities cannot be derived about the proximate mechanisms which regulate ageing. Indeed, some evidence exists that the mechanisms underlying exceptional longevity may be consistent across taxa (Treaster et al. 2021). Different evolutionary patterns of ageing clearly exist; thus, the question becomes what are the universal proximate drivers of senescence which might exist in all species regardless of how ageing evolved.

**Fig. 1:**
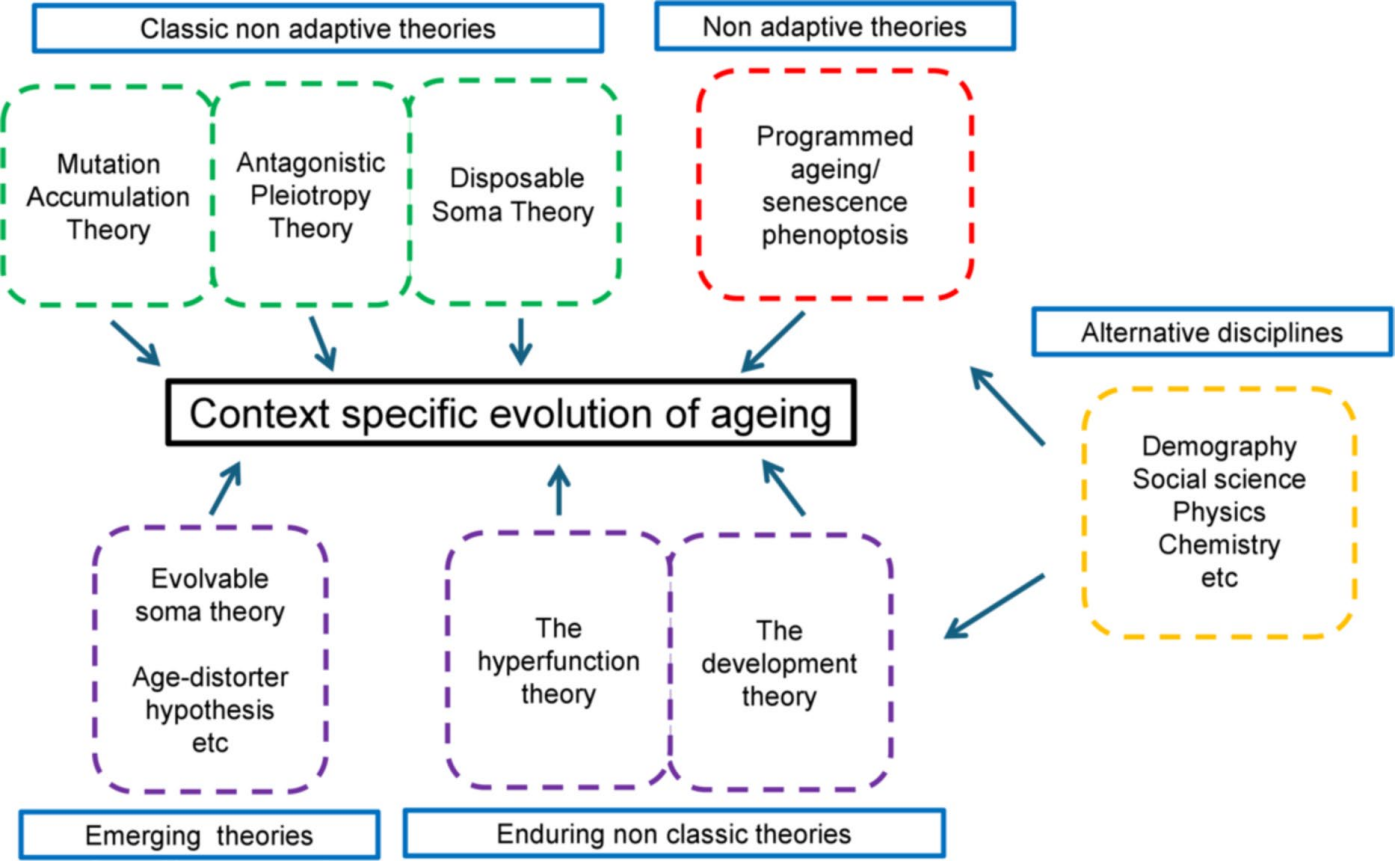
The evolution of ageing: a pluralistic framework. Within this framework it is acknowledged that different models are appropriate for different taxa and depend on the ecological context. This version of pluralism also recognises the important role other disciplines, including physics and social science can contribute to our understanding of ageing

Figure 1 illustrates the proposed framework. Each theory has the potential to account for the evolution of ageing, however some are more suited to explaining the evolution of ageing within certain ecological contexts. It underscores that the classic theories are not mutually inclusive and may operate simultaneously or synergistically to bring about different evolutionary patterns of ageing. Thus, in most cases their expansion is not necessary, and it is merely important to recognise that one single model does not describe the evolution of ageing across the tree of life. Indeed, convincing evidence exists for the evolution of lifespan being a species specific trait (Shilovsky et al. 2022). For instance, based on the pluralistic framework outlined in Fig. 1 an explanation of primate ageing might involve different aspects of the classic theories. To this end MA and AP are well supported logical models which provide a coherent explanation as to why certain genetic polymorphisms such as APOE4 are linked with ageing, while arguably DS provides a reasonable explanation of the nexus between DR and the evolution of ageing in primates.

This framework also includes alternatives to the classic theories. This is important because complimentary ideas, such as virtual gerontogenes, ELS and homeodynamic space can be assimilated within classic evolutionary thinking. The inclusion of virtual gerontogenes recognises that ageing genetic entities can exist that were not originally selected as genes/alleles either for or against ageing. Moreover, a pluralistic approach which, provides scope for the inclusion of ideas around ELS and homeodynamic space acknowledges that the evolution of ageing is a dynamic, emergent and ultimately malleable process (Chmielewski 2020). At the same time a pluralistic approach provides scope for the inclusion of adaptive evolutionary arguments. However, it is necessary to acknowledge that it is unlikely ageing is genetically programmed simply because it proceeds towards a final state in a predictable fashion. Such an observation may simply indicate the biological system is built in a particular way and so always fails in more or less the same manner each time, due to thermodynamic constraints.

Figure 1 illustrates that it is important to consider the role of other disciplines such as mathematics, physics, chemistry, demography and social science can play in improving our understanding of ageing from a pluralistic perspective. Applying physical principles to biology can generate novel insights (Kinsey et al. 2023). As outlined in section "Darwinian fitness" statistical thermodynamics has been effectively applied to age structured populations in the form of EE, which offers a more concrete way of representing Darwinian fitness. Recognising that senescence and death are subject to the laws of thermodynamics, enables EE to be used within a pluralistic framework. If this is the case then the force of natural selection becomes a function of age (Demetrius and Ziehe 2007). And, including EE, helps to overcome a major limitation of the classic theories, namely that they do not include population size as a critical component of the process of selection (Dietz 2005; Brink et al. 2009). Moreover, the models MA, AP and DS all invoke the same measure of fitness. Thus, the theories based on MA, AP, and DS can be considered as limiting cases of the theory based on EE, and their relationship with EE serve to emphasize its universality (Demetrius and Legendre 2013). Consequently, a pluralistic model-based around EE as a measure of Darwinian fitness has the potential to be more expansive than the classic models of ageing on their own, which utilise the Malthusian parameter as a measure of Darwinian fitness. The importance of EE as a measure of Darwinian fitness cannot be understated as a concept, in fact it would be worthwhile to see it combined with other recent ides such as an idea proposed by Lissek and colleagues which suggested that adaptive processes e.g. transcription, epigenetic remodelling, and metabolic plasticity drive maladaptation and cause the diseases of ageing (Lissek 2024). Known as the adaptation-maladaptation theory, this idea resonates with thermodynamic thinking, as at its core is the notion that adaptive processes dysregulate biological systems.

## Discussion and conclusions

Over the millennia considerable thought has been invested into understanding why organisms age. However, it was only when natural selection was introduced that a meaningful theoretical framework could be used to solve this problem. Natural selection forms the core of three classic evolutionary theories which have attempted to explain why this process occurs. The three classic theories are competing yet not mutually exclusive as they assume that ageing is the result of the declining efficacy of natural selection. These theories have endured for over half a century. In this time a reasonable body of experimental data has been identified which supports various aspects of the classic theories. However, emerging evidence has called into question the suitability of the classic theories. Considering this the overarching aim of this paper was to discuss the classic theories from the perspective of their limitations. A further objective was to discuss the classic theories in light of emerging theoretical alternatives. The final goal of the paper was to introduce a more pluralistic view of the evolution of ageing.

In terms of examining the limitations of the classic theories this work uncovered some noteworthy problems which present genuine theoretical and experimental challenges for the classic theories. It was outlined how using EE is potentially a more effective way of representing Darwinian fitness than using the Malthusian parameter. Circumstances were also highlighted whereby an increase in extrinsic mortality may not result in the evolution of decreased lifespan. It was also revealed that negative/neutral senescence is difficult to reconcile with the classic theories. Moreover, it was illustrated how the interplay between natural and sexual selection can impact the evolution of senescence in both males and females. However, it is crucial to stress that significant controversy remains over the role many of these issues play within classic evolutionary thinking.

Arguably a shortcoming of this review is that it did not outline every conceptual difficulty currently faced by the classic theories. Undoubtably this would have resulted in an extended manuscript, which may have failed to capture the key challenges to the classic theories. Despite this however, it is worth briefly mentioning some mechanisms which were not discussed that intersect with the challenges explored in this work. For instance, the suggested pleiotropic action of genes featured prominently in our discussion of AP. However, antagonism is merely one form of pleiotropy (Zhang 2023). Pleiotropy comes in many guises. An example is positive pleiotropy, which is considered to influence the evolution of ageing (Maklakov et al. 2015). A compelling case of positive pleiotropy can be found in *Drosophila* where the Indy mutation is associated with both a slower ageing and a normal fecundity rate (Marden et al. 2003). Similar examples of positive pleiotropy exist in other canonical organisms used in ageing research. Moreover, an area that requires careful consideration in future is how different forms of pleiotropy have influenced the evolution of ageing. However, this is a complex undertaking which will perhaps only be solved by using computational analysis. Computational approaches have proved effective recently at elucidating other complex problems in evolution such as primate evolution (Shao et al. 2023). Computational analysis is relevant to another point raised in this review. It was revealed that the classic models do not consider how animal–microbiome interactions may have influenced the evolution of ageing. The inherent biological complexity of microbiomes presents a significant challenge to the classic theories. It has been suggested that it is necessary to integrate the microbiome into quantitative genetics to help disentangle the complexities of host-microbiome evolution (Henry et al. 2021). This is important for any theoretical interpretation of the evolution of ageing, as it suggests that dynamic genetic multidirectional symbiotic ensembles are an important evolutionary force.

The issues raised above together with the difficulties presented in the main body of this review are challenging for the classic theories to solve. This is the reason the final section of this review was devoted to advocating for a more pluralistic interpretation of the evolution of ageing. This version of pluralism introduced here is conceptually close to the emerging field of biodemography which recognises the importance of an interdisciplinary approach to understanding the evolutionary origins of disease (Hernández-Pacheco et al. 2023). It also emphasises the importance of using EE as a measure of Darwinian fitness as opposed to relying on models which depend on the Malthusian parameter. Such an approach is needed to better understand the evolution of ageing. The rationale for introducing this pluralist approach was to infuse the evolution of ageing with a more fluid framework, given that the evolution of different rates of ageing are very much species and context dependent. However, it is worth acknowledging that pluralism may not be the best way to gain a better understanding of the evolution of ageing. It is theoretically possible the classic theories, and their alternatives can be reduced to a final unified theory. Indeed, given that nascent theories continue to emerge this could be a possibility. In the meantime, however, pluralism represents a flexible alternative which can accommodate both classic and emerging ideas.

## Data Availability

No datasets were generated or analysed during the current study.
